# Supplementary material for: Rat and mouse cardiomyocytes show subtle differences in creatine kinase expression and compartmentalization
Source: PLoS One. 2023 Nov 27;18(11):e0294718. doi: 10.1371/journal.pone.0294718 (PMC10681188; doi:10.1371/journal.pone.0294718)
Supplement: S1 Table — The maximal respiration rate, VO2_max, was recorded in the presence of either GM or GMPS, and 2 mM ADP (see representative recording in Fig 3A). The rate was normalized to the protein content and, for rat cardiomyocytes only, the cytochrome aa3 content. For statistical purposes, only the results from CK recordings are given. The corresponding maximal ADP-phosphorylation rate, VADP_max, was calculated assuming P/O2 ratios of 6 and 4 for GM and GMPS, respectively (see main text). Values from 8 mice and 7 rats are shown as mean ± SEM. * denotes p < 0.05, ** p < 0.01, *** p < 0.001, **** p < 0.0001, significant effect of substrate, species, or interaction between substrates and species. (DOCX) [file pone.0294718.s001.docx]

# Supplementary Information for:

# Rat and mouse cardiomyocytes show subtle differences in creatine kinase expression and compartmentalization

Jelena Branovets, Kärol Soodla, Marko Vendelin, Rikke Birkedal^*^

**S1 Table. Maximal respiration rate, V_O2_max_, and maximal ADP-phosphorylation rate, V_ADP_max_, normalized to the protein content of the cell suspensions, and V_O2_max_, normalized to the cytochrome aa_3_ content.**

|  | | **V_O2__max/protein** | **V_ADP__max/protein** | **V_O2__max/cyt aa3** |
| --- | --- | --- | --- | --- |
|  | | nmol O_2_ /min/mg protein | nmol ADP /min/mg protein | nmol O_2_ /min/cyt aa_3_ |
| Mouse | GM | 48.8 ± 1.9 | 293 ± 12 |  |
|  | GMPS | 99.6 ± 4.0 | 399 ± 16 |  |
| Rat | GM | 43.8 ± 2.5 | 263 ± 15 | 272.8 ± 18.5 |
|  | GMPS | 78.2 ± 5.2 | 313 ± 21 | 482.0 ± 23.6 |
| Substrate | | **** | **** | **** |
| Species | | ** | ** |  |
| Substrate x species | | * | * |  |

The maximal respiration rate, V_O2_max_, was recorded in the presence of either GM or GMPS, and 2 mM ADP (see representative recording in Fig. 3A). The rate was normalized to the protein content and, for rat cardiomyocytes only, the cytochrome aa_3_ content. For statistical purposes, only the results from CK recordings are given. The corresponding maximal ADP-phosphorylation rate, V_ADP_max_, was calculated assuming P/O_2_ ratios of 6 and 4 for GM and GMPS, respectively (see main text). Values from 8 mice and 7 rats are shown as mean ± SEM. * denotes *p* < 0.05, ** *p* < 0.01, *** *p* < 0.001, **** *p* < 0.0001, significant effect of substrate, species, or interaction between substrates and species.
