# Supplementary material for: Rat and mouse cardiomyocytes show subtle differences in creatine kinase expression and compartmentalization
Source: PLoS One. 2023 Nov 27;18(11):e0294718. doi: 10.1371/journal.pone.0294718 (PMC10681188; doi:10.1371/journal.pone.0294718)
Supplement: S1 Raw images — (PDF) [file pone.0294718.s003.pdf]

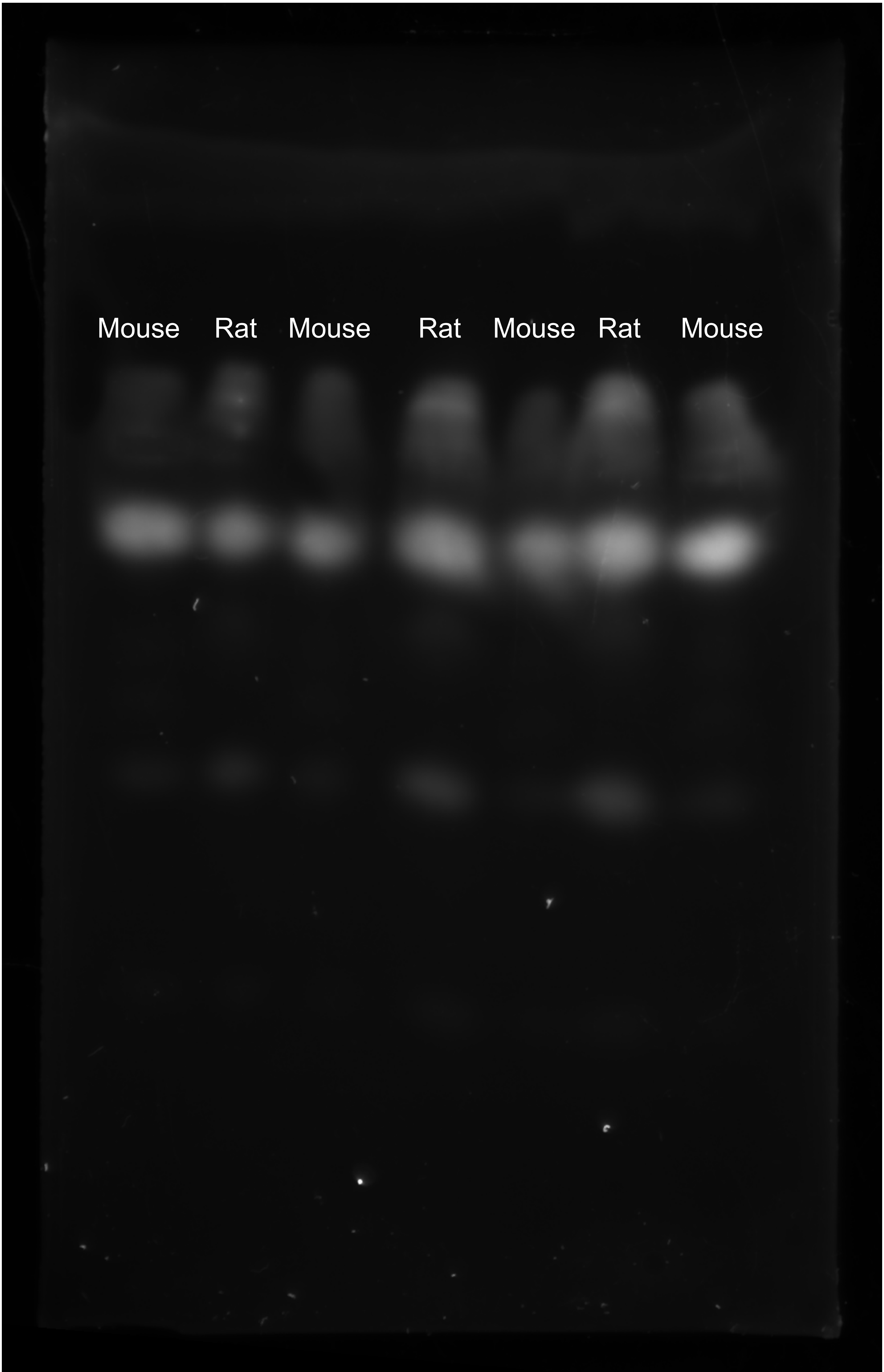
Mouse
Rat
Mouse
Rat
Mouse
Rat
Mouse

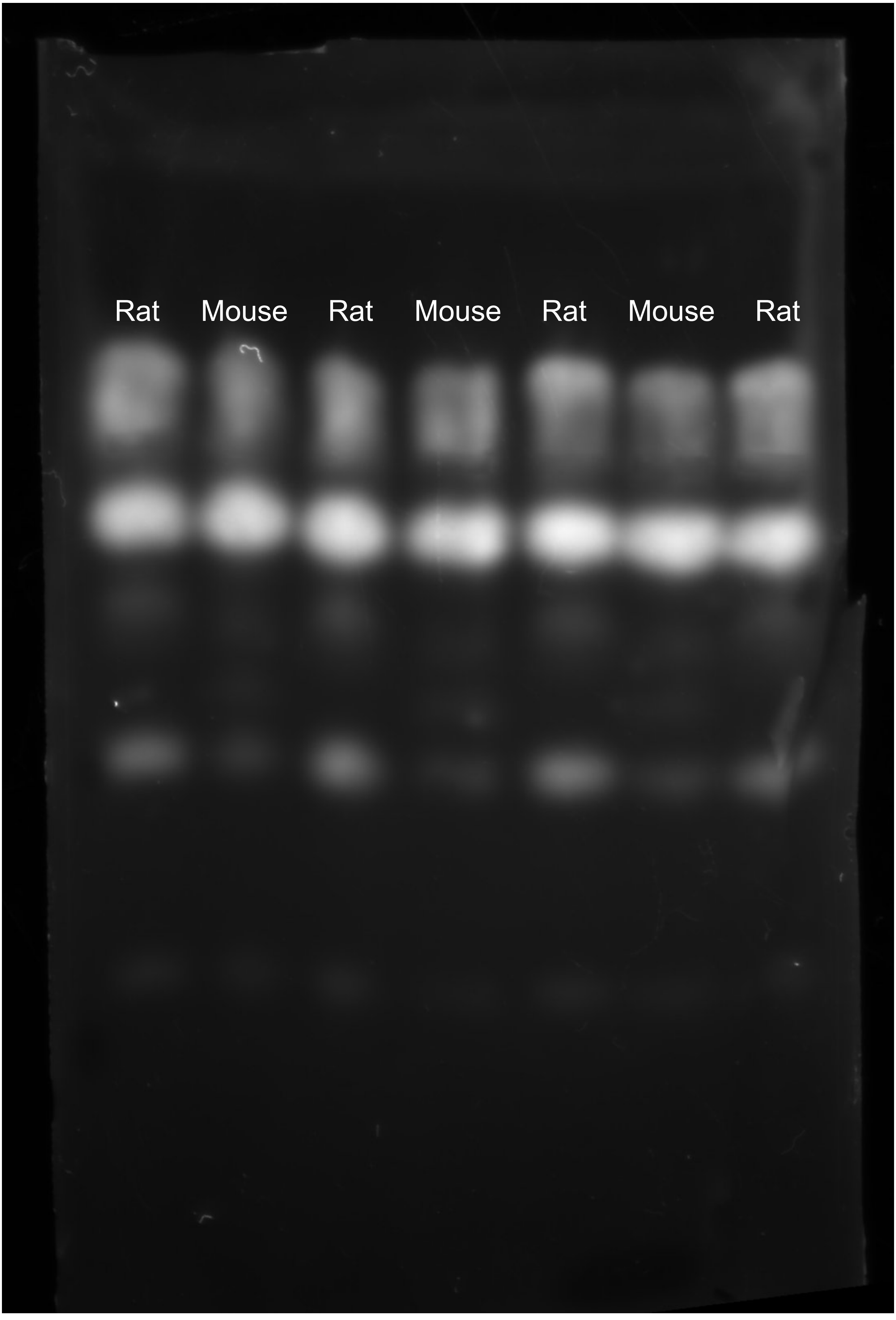
Rat
Mouse
Rat
Mouse
Rat
Mouse
Rat

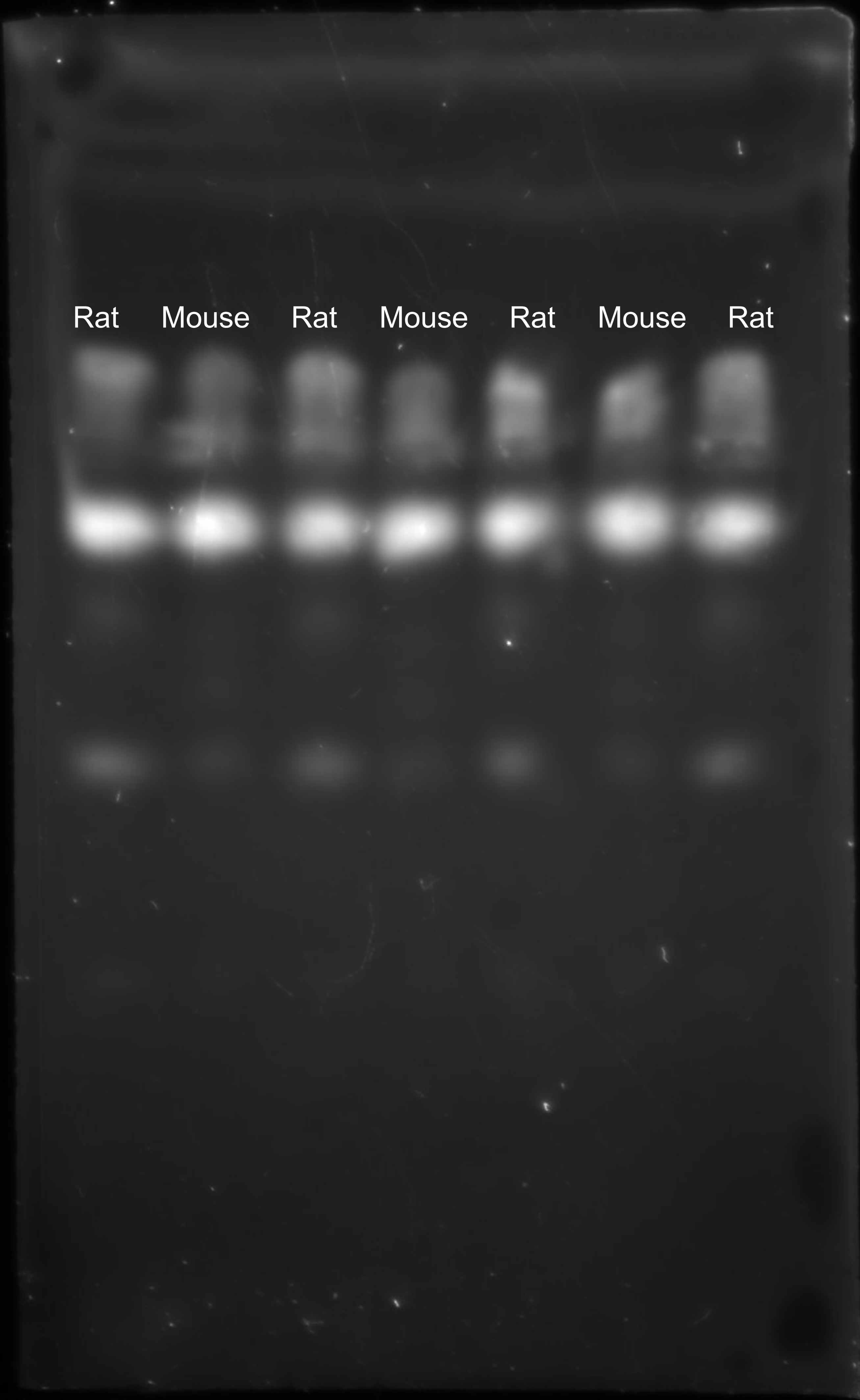
Rat
Mouse
Rat
Mouse
Rat
Mouse
Rat
