# Supplementary material for: Rat and mouse cardiomyocytes show subtle differences in creatine kinase expression and compartmentalization
Source: PLoS One. 2023 Nov 27;18(11):e0294718. doi: 10.1371/journal.pone.0294718 (PMC10681188; doi:10.1371/journal.pone.0294718)
Supplement: Attachment — Submitted filename: Review Marks.docx [file pone.0294718.s004.docx]

**Review for PLOS ONE	09/06/2023**

**Title: [“Rat and mouse cardiomyocytes show subtle differences in creatine kinase expression and](http://review.frontiersin.org/Document/DownloadPDF?articleId=624704&siteId=188&userId=251194&roleId=13)**

**[compartmentalization”](http://review.frontiersin.org/Document/DownloadPDF?articleId=624704&siteId=188&userId=251194&roleId=13)**

The manuscript described the subtle differences in CK expression and compartmentalization between rat and mouse cardiomyocytes. The methodologies are clear and sound, with convincing data.

There are some concerns about the manuscript in its present form:

1. Since the whole heart homogenates and isolated cardiomyocytes might have different characterizations, please explain the possible difference(s) and how the two could be integrated together.
2. There are extensive similar (or same) descriptions amongst the main text, figure legends, and method section. It would be better to integrate them to make the manuscript clear and concise.
3. Since the extensive usage of abbreviations, please move the Table of Abbreviations to the front, and define the abbreviations when they appear the first time. Also, please add Cyt aa3 to the list.
4. Line 59-60: Please add the reference and characterization of AGAT and GAMY mouse strains.
5. Line 142-145: Did the enzymatic activities normalize to the “heart” wet weight?
6. Fig 2B: The BB-CK bands are hardly recognized. It might be helpful to enhance the image.
7. Line 246: Please define P/O2 value.
8. Line 750: “Drops of 1ul homogenate”, does the homogenate normalized to the same protein concentration?
9. Please add a paragraph to explain the normalization method(s) that make the measurements are comparable between mouse and rat model.
