# Supplementary material for: Rat and mouse cardiomyocytes show subtle differences in creatine kinase expression and compartmentalization
Source: PLoS One. 2023 Nov 27;18(11):e0294718. doi: 10.1371/journal.pone.0294718 (PMC10681188; doi:10.1371/journal.pone.0294718)
Supplement: Attachment — Submitted filename: Branovets et al revised Response to reviewers.docx [file pone.0294718.s005.docx]

Response to reviewers

We would like to thank the reviewer for his/her constructive comments, which have improved the manuscript. We have addressed the comments point-by-point in the text below. For clarity, the reviewer comments are in italic and our response is in normal font.

*The manuscript described the subtle differences in CK expression and compartmentalization between rat and mouse cardiomyocytes. The methodologies are clear and sound, with convincing data.*

*There are some concerns about the manuscript in its present form:*

1. *Since the whole heart homogenates and isolated cardiomyocytes might have different characterizations, please explain the possible difference(s) and how the two could be integrated together.*

Indeed, isolated cardiomyocytes are different from whole heart homogenates, as the latter also contains extracellular matrix, endothelial cells and fibroblasts. In order to compare the two different preparations, we assumed that the citrate synthase (CS) activity in non-cardiomyocytes is negligible and compared the data normalized to the CS activity. To explain this, we have inserted a paragraph at the end of the Introduction in lines 121-129.

2. *There are extensive similar (or same) descriptions amongst the main text, figure legends, and method section. It would be better to integrate them to make the manuscript clear and concise.*

Thank you for this comment. We have shortened the legends of Figures 3 and 4 accordingly – see lines 224-236 and 341-351, respectively – as well as the description in the Methods – see lines 656-665.

3. *Since the extensive usage of abbreviations, please move the Table of Abbreviations to the front, and define the abbreviations when they appear the first time. Also, please add Cyt aa3 to the list.*

The table of abbreviations has been moved to the front and Cyt aa_3_ has been added to the list. Furthermore, we checked all the abbreviations and made sure that they are defined when they appear for the first time.

4. *Line 59-60: Please add the reference and characterization of AGAT and GAMY mouse strains*.

We have added a reference for AGAT and GAMT mice (Choe et al., 2013, and Schmidt et al., 2004, respectively). They are now in lines 62-63.

5. *Line 142-145: Did the enzymatic activities normalize to the “heart” wet weight?*

Thank you for catching this. Yes, we have inserted “heart” to emphasize that the activities were normalized to the heart wet weight. This is now in line 153.

6. *Fig 2B: The BB-CK bands are hardly recognized. It might be helpful to enhance the image.*

Indeed, BB-CK bands are hardly visible. Unfortunately, we could not enhance the pseudocolor image further, as we have to comply with the image enhancement requirements. In particular, enhancing the image further will saturate the region corresponding to MMCK. As an extra note, the figure quality was reduced during the manuscript PDF generation by the journal. The quality is better in the original figure that is available to download through the link at the top right of the page with the figure in PDF.

7. *Line 246: Please define P/O2 value*.

This has been defined in the text (now in line 250) as well as in the list of abbreviations.

8. *Line 750: “Drops of 1ul homogenate”, does the homogenate normalized to the same protein concentration?*

For the electrophoresis of CK isoforms, we used the same homogenates as for the recordings of enzyme activities, which were all at a concentration of 50 mg wet weight/ml homogenate. We did not measure protein concentration of these homogenates, because they contain Triton X-100, which interferes with the spectrophotometric recording of protein content.

To clarify the text, we have added in lines 737-738 that 1 μl homogenate corresponds to tissue extract from 50 μg heart wet weight.

9. *Please add a paragraph to explain the normalization method(s) that make the measurements are comparable between mouse and rat model.*

For the whole tissue homogenates, the enzyme activities were normalized to the wet weight of the tissue, allowing a straightforward comparison between rat and mouse (Figure 1A). In addition, the homogenate (Figure 1B) and respiration data (Figures 3 and 4) were normalized to the CS activity because (1) this correlated with the respiration capacity and our research question was how the rate of ADP generated by CK or AK is related to the respiration capacity in rat and mouse, and (2) it allowed us to compare whole heart homogenates and isolated cardiomyocytes as explained above.

We have expanded our earlier justification of use of CS activity (lines 121-129) by adding a paragraph (lines 386-392) to link with your concern #1 and stress that it can be used to relate activities across species.

We hope that we addressed this concern as you expected.
